# Platforms, advances, and technical challenges in virus-like particles-based vaccines

**DOI:** 10.3389/fimmu.2023.1123805

**Published:** 2023-02-09

**Authors:** Reeshu Gupta, Kajal Arora, Sourav Singha Roy, Abyson Joseph, Ruchir Rastogi, Nupur Mehrotra Arora, Prabuddha K. Kundu

**Affiliations:** Department of Research and Development, Premas Biotech Pvt Ltd., Sector IV, Industrial Model Township (IMT), Manesar, Gurgaon, India

**Keywords:** virus like particles, enveloped VLP, non-enveloped VLP, chimeric VLP, vaccine, recombinant DNA technology

## Abstract

Viral infectious diseases threaten human health and global stability. Several vaccine platforms, such as DNA, mRNA, recombinant viral vectors, and virus-like particle-based vaccines have been developed to counter these viral infectious diseases. Virus-like particles (VLP) are considered real, present, licensed and successful vaccines against prevalent and emergent diseases due to their non-infectious nature, structural similarity with viruses, and high immunogenicity. However, only a few VLP-based vaccines have been commercialized, and the others are either in the clinical or preclinical phases. Notably, despite success in the preclinical phase, many vaccines are still struggling with small-scale fundamental research owing to technical difficulties. Successful production of VLP-based vaccines on a commercial scale requires a suitable platform and culture mode for large-scale production, optimization of transduction-related parameters, upstream and downstream processing, and monitoring of product quality at each step. In this review article, we focus on the advantages and disadvantages of various VLP-producing platforms, recent advances and technical challenges in VLP production, and the current status of VLP-based vaccine candidates at commercial, preclinical, and clinical levels.

## 1 Introduction

Traditional vaccines, such as inactivated or live-attenuated, have shown protective efficacy against several infectious diseases that are caused due to viral infections. However, they sometimes have safety concerns and deviations in the protective immune response. Recent advances in nucleic acid-based, subunit, and recombinant viral vector-based vaccines may overcome the limitations of traditional vaccines against infectious pathogens by enhancing immunity without compromising safety, efficacy, and tolerability and can also control infectious diseases during outbreaks or pandemics. The significance of such vaccine approaches in rapidly developing vaccine candidates for pandemic threats has been previously reported and is considered an approach for pandemic preparedness and response (1). Virus-like particles (VLP) differ from other subunit vaccines in that they have strong immunogenicity because they present repetitive antigenic epitopes to the immune system in more authentic confirmation and move in parallel with viral vector-based vaccines to produce protective, effective, and efficient vaccines against severe infectious diseases (2).

VLPs are non-infectious multiprotein structures that mimic native viruses but lack the disease-causing viral genome, making them safer vaccine candidates. Because of their optimal size (20–200 nm), they can practically display any epitope in a multivalent format that guarantees a high humoral or cell-mediated immune response in the host.

VLPs are categorized into enveloped and non-enveloped based on the presence or absence of lipid envelopes. Furthermore, these particles can be classified as homologous or heterologous according to their composition. Homologous VLPs contain proteins derived only from the native viruses. On the other hand, heterologous VLPs contain proteins from different sources, which increases their immunogenicity (3). They are further classified as single- or multi-capsid proteins. Simple VLPs are composed of a single capsid protein that is expressed in both eukaryotic and prokaryotic systems. In contrast, multi-capsid proteins show several striking structural characteristics, including many complicated concentric layers of different capsid proteins. Multi-capsid VLPs are usually produced in eukaryotic expression systems such as yeasts, insect cells, and plants. For example, various combinations of four different rotaviral capsid proteins can produce stable VLPs with double or triple capsid layers (4). Complex multi-capsid VLPs present higher production challenges. In this case, each protein should be expressed in stoichiometric amounts for efficient production because of limited cellular resources, as the expression of excess monomers does not improve VLP assembly (5). Enveloped VLPs (eVLPs) are poorly characterized at the biophysical level because they are structurally less uniform than non-enveloped VLPs. Technical challenges remain in the generation of eVLP-based vaccines in their design, treatment, and storage. Proper folding and glycosylation of viral surface antigens are important for defining the efficacy of eVLP-based vaccines because they are critical for stability, immune recognition, and pathogenicity. In general, eVLPs with host-derived envelopes are more sensitive to the external environment than non-enveloped VLP. Changes in conditions, such as temperature, shear force, and purification processes, can destroy the integrity and stability of particles, reducing eVLP immunogenicity. However, computational design, *in vitro* assembly of VLPs, removal or replacement of the transmembrane region, introduction of a suitable heterologous signal peptide, new bioprocessing modalities, and new macro-scale purification materials can address these problems robustly, at the expense of process complexity (6).

VLPs carry pathogen associated molecular patterns (PAMP), which are sensed by pathogen recognition receptors (PRRs) present either on the cell surface or endosomes of dendritic cells (DC). Presentation of VLP-peptides along with major histocompatibility complex (MHC) class I or class II molecules by DC induces CD8+ or CD4+ T cell responses, respectively (7). Simultaneously, co-stimulatory molecules present on DC activate T-helper (Th) cells, which are required for both cell-mediated and humoral immunity. In some cases, B cells are activated without innate immunity or any direction by Th cells (6). The addition of more immunogenic domains in VLP could further enhance their potency and efficacy, as seen in the case of hepatitis B core antigen (HBcAg). HBcAg-based VLP vaccines contain more immunogenic domains of hepatitis B surface antigen (HBsAg) and stimulate broad and specific humoral and cell-mediated responses against hepatitis B virus (HBV) (8). Because VLPs elicit a protective response at lower doses, they can significantly reduce vaccine costs (9).

Several platforms have been used for VLP production. The choice of the preferred platform provides flexibility in the manufacturing conditions required for the scaled-up production of VLP vaccines (7). Remarkably, VLPs can be recombinantly produced by expressing either a single type of coat protein, multiple structural proteins, or a combination of structural and nonstructural proteins required for VLP production. Examples include the first commercial VLP-based vaccine licensed for the HBV (9). To date, hundreds of VLP-based vaccine candidates have been studied for immunization against various human and animal diseases. However, only a few have been commercialized to target non-enveloped viruses. The structure of non-enveloped VLPs consists of either a single or multiple structural protein(s) of a specific virus and is relatively simple. In contrast, eVLPs have host cell components that pose potential challenges to their regulatory approval, scalability, and stability (10). Both types of VLPs can be engineered to form chimeric VLP, which display foreign antigens to enhance immune response and can also be used as nanocarriers for the delivery of desired antigens or therapeutic component of a molecule (11).

This article briefly presents the merits and demerits associated with various platforms, recent advances, and technical challenges in VLP production. The current scenario of VLP-based vaccine candidates at commercial, clinical, and preclinical levels are also summarized.

## 2 Expression platform for VLPs

VLP-based vaccines have been developed as the next-generation vaccines. However, to ensure optimal protein folding, post-translational modifications (PTMs), cost-effectiveness, and scale-up production of these vaccines in a timely manner, an appropriate platform must be selected. VLPs can be experimentally generated in the laboratory using recombinant viral proteins that are expressed in a range of expression systems including prokaryotic cells, yeast, insect cell lines, plants and mammalian cell lines. In addition, cell-free expression techniques have been used successfully. In these cases, VLP proteins are expressed first in a cell-based expression system, then assembled in a cell-free environment for proper folding (6). All systems have their own advantages and drawbacks, which are briefly listed in **Table 1**.

**Table 1:** Merits and demerits of various platforms of VLPs.

Platform	Merits	Demerits
Bacteria	1)Least expensive 2)Easy to use 3)Fast growth rates; High yield 4)High scalability 5)High level expression	1)Lacks post-translational modification (PTM), thus produce simple VLPs 2)Presence of host-cell derived components and, 3) difficulty to express soluble and full-length products create a potential bio-separation problem and an increase in final production cost
Yeast	1)Less expensive 2)High density fermentation 3)Modification of the expression platform 4)Moderately rapid expression 5)Supports protein folding and PTM 6)Low risk of contamination during scale up	1)High Mannose modification i.e. yeast N glycosylation patterns are of a high mannose type, which is immunogenic and reduces recombinant protein serum half-life in mammals (12) 2)Enhanced safety precautions 3)No ideal results in case of several secretary proteins with a high molecular mass
Insect	1)Moderately rapid expression 2)Supports protein folding and PTM 3)Works well for both non-enveloped and enveloped VLPs, free of mammalian pathogens	1)High cost 2)Low scalability 3)Simpler N glycosylation, 4)Contamination with baculovirus particles 5)Low level expression 6)Complex downstream processes
Mammalian	1)Works well for enveloped and non-enveloped viruses 2) Appropriate PTM 3) Authentic assembly and folding of recombinant proteins	1) High cost 2) Difficult to scale-up 3)Time consuming 4) Low yield 5) Susceptible to infection to mammalian pathogens
Plant	1)Rapid expression 2) Highly scalable 3) Low cost of up and downstream processing	1)Low yield 2)Regulatory and technical issues 3) Antigen degradation during *in vivo* delivery 4)Inability of proteins to undergo assembly, folding, stability and PTMs 5) Simple structure (one recombinant protein)
Cell-free expression system (6)	1) Time-saving 2) High yield of proteins 3) Limited cellular contaminants 4) The option of producing VLPs containing unnatural amino acids (UAAs) or toxic protein intermediates	1) Very high production cost 2) Limited scalability

The optimal expression of each VLP is usually identified *via* trial-and-error by comparing the translated products of multiple expression systems. Expression in *E. coli* is often preferred for producing small proteins with limited PTMs; however, PTMs of larger proteins require a more complex expression system, such as yeast, baculovirus, and mammalian cells. Compared to higher eukaryotes, yeasts are considered a cost-effective production system. In addition, yeasts avoid endotoxin and viral contamination problems associated with bacterial and mammalian expression systems (13). Engerix-B (HBV vaccine) and Gardasil (Human Papilloma Virus (HPV) vaccine) are two FDA-approved vaccines that have been generated in yeast expression systems. Despite these achievements, the lack of complex PTMs is a major drawback of yeasts, which limits their use in generating non-enveloped VLPs. In contrast, animal cell expression platforms are attractive because of their ability to make complex and precise PTMs that are essential for proper protein folding, which can be used to produce multiple structural proteins of non-enveloped and enveloped VLPs (6).

Due to the ease and speed of baculovirus-based VLP expression, this system is suitable for manufacturing vaccines against viruses that rapidly change their surface antigens between outbreaks, such as influenza virus (6, 14). Cervarix is an FDA-approved HPV vaccine produced using this expression system. The baculovirus/insect cell platform has also been used as a prophylactic vaccine candidate against several infectious diseases such as human immunodeficiency virus (HIV) 1, influenza virus A, chikungunya virus, severe acute respiratory syndrome (SARS), Ebola virus, dengue fever virus, Rift Valley fever virus (RVFV), Norwalk virus, and hepatitis C virus (HCV) (6, 15). The major drawback of the baculovirus/insect cell platform is its simpler N-glycosylation pattern compared to that of mammalian cells, which can be a disadvantage for some VLP applications.

VLP production in transgenic plants has several interesting applications. For example, although plant cells lack mammalian-like PTMs, plant-specific glycosylation can have an immunostimulatory effect (13). Plants have been used for VLP production of the Norwalk virus (16, 17), HIV-1 (18), and influenza virus VLPs (Medicago). Another recently developed expression platform is the cell-free system, which usually consists of extracts from *E. coli* or yeast cells. This system is preferred for the production of viral capsid proteins with toxic intermediate forms. Owing to the non-replenishing nature of a cell-free system, this method is highly demanding, with scalability limitations. Examples of commercialized VLP vaccines produced in a cell-free expression system include the influenza vaccine Inflexal V (19) and the hepatitis A vaccine Epaxal (20).

## 3 Culture mode used for the production of multimeric VLP

Three culture modes were used to produce the VLPs: batch, fed-batch, and continuous cultivation. Process design is of crucial importance because it influences yield, productivity, and final product concentration. The cost of raw materials can be decreased by maintaining a yield close to the maximum theoretical value. Simultaneously, an increase in high product concentration and productivity, and a reduction in non-productive periods can reduce the cost of downstream processing and contribute to the efficient use of manufacturing equipment. Owing to its simplicity and low risk of contamination, the batch mode is preferred for many industrial processes. In batch culture, all media components are added to the bioreactor at the start of the cultivation process, with no extra nutrients supplied until the end. However, the maximum potential of this process is often not achieved. Substrate and product inhibition, low virus titers, temperature sensitivity, low productivity (21) and ineffective use of time between batches are major problems in the batch mode. For efficient performance of batch fermentation, all non-productive steps (such as cleaning and filling of bioreactor, media formulation, etc.) should be minimized while targeting the maximum yield of the end product (22). This approach has been used to test a variety of VLPs, including HIV (23), chikungunya virus (24) and Ebola (25). In a fed-batch process, substrate inhibition is avoided by reducing the lag phase, while correctly designed feeding of concentrated nutrients increases product concentration and yield. The rate of the reaction is determined by the amount of limiting nutrients in the feed medium. In a baculovirus/insect cell expression system, this culture mode was tested for the production of parvovirus-like particles (26) and recombinant HBsAg (27). The highest productivity is achieved in continuous mode because the culture is in the log phase. In this mode, fresh medium is supplied and the conditioned medium is extracted. Continuous production is obtained using this method, and the product must be stored under proper conditions while being produced. This approach has been used for producing rabies VLPs in HEK293 cells (28). There are many types of continuous modes such as chemostat, turbidostat, stressostat, and morbidostat. Chemostat is the most widely used continuous cultivation method (29). It is possible to control the specific growth rates and product formation in a chemostat by changing the concentration of the limiting substrate for a specific strain. However, the increased risk of contamination, high volumes of low-concentration broth from the system, and low product concentrations are problematic (30). The fermentation process should be selected by considering the advantages and disadvantages of each process. The major criterion for the selection of a bioreactor/fermentation process is the minimal capital cost per unit product yield. For example, the design of a continuous production process for measles virus is more challenging because of its size (300 nm – 1 μm) and lysis of host cells. Cell lysis leads to rapid accumulation of host cell proteins (HCPs) and cell debris in the surrounding medium. Therefore, filtration during cultivation is challenging because the filter pores are blocked by debris or HCPs. Discontinuous filtration or repeated batch mode is more appropriate for this virus (21, 31). The corresponding yields from the different culture modes are summarized in **Table 2**.

**Table 2:** Yield associated with the various culture modes using different VLP platforms.

Virus	Expression platform	Culture mode	Yield (Bulk)	Yield (Purification)	Reference
Foot-and Mouth disease virus	*E. coli*	Fed Batch	~50mg/L		(32)
Rabbit hemorrhagic disease virus	*Pichia pastoris*	Seed-Batch		2–2.5 million doses (0.05g/l)	(33)
Hand, foot and mouth disease virus (Enterovirus)	*Pichia pastoris*	Batch (Bioengineering F22 fermenter)		0.13mg/g	(34)
HIV	CHOEBNALT85 cell line (QMCF/CHO-based stable cell line. QMCF technology utilizes genetic modifications which increase the expression vector copy-number while ensuring their even distribution during cell division, guaranteeing near-stable expression levels)	Monoliths		0.131g/l	(35)
Porcine circovirus	*E. coli*	Shake Flask		50g/l bacterial culture	(36)
Adeno Associated Virus (AAV)	Baculovirus	Shake Flask and Bioreactor culture	18 μg/ml		(37)
Chikungunya	Baculovirus	Larger shake flasks and stirred tank bioreactors	28mg/L		(38)
Influenza	Baculovirus	Shake and spinner flask cultures	512 HAU/50 μl		(39)
Rotavirus	Yeast	Fed Batch culture	Volumetric and specific productivities increased 28.5- and 11-fold, respectively, in comparison with batch cultures		(40)
SARS-CoV-2	Bacteria	Batch		0.25g/l	(41)

The bioreactors used in the manufacturing of VLP-based vaccines are made of stainless-steel vessels. However, single-use technology is gaining importance since it eliminates the need for *in-situ* cleaning and sterilizing, which reduces cross-contamination, investment, and operational costs. However, single-use vessels are less useful (14). Each of the three culture modes have several advantages and disadvantages (**Table 3**).

**Table 3:** Advantages/challenges associated with the various culture modes used for the production of VLPs.

Culture mode	Advantages	Challenges
**Fed-Batch**	Extend the exponential growth phase by adding selective addition of nutrients and/or amino acids Extend the duration of culture for higher cell densities or switch metabolism to produce recombinant protein (42)	Scale-up becomes expensive since culture medium is inefficiently used
**Batch**	Medium is well utilized, and the product is highly concentrated	The growth rate is slower because nutrient levels decline with time. Batch cultures are less efficient as the fermenter is not in operation all the time.
**Continuous**	Short ‘turn-around’ time (i.e., reduces the cost associated with cleaning and filling of the bioreactor) and a small number of production steps. Most downstream processing operations are performed in a continuous manner, fitting well with overall operation of a bioprocess plant.	Most of these systems require a two-stage configuration (cell growth and infection) and generate high amounts of defective interfering particles (infectious viruses lacking complete viral genomes) when operating for long periods of time. The complexity of the bio-product produced is also generally low. The need of regulatory agencies to link a production batch to a specific biopharmaceutical product for its approval and licensing has been hindering a broader generalization of continuous cultures.
**Perfusion mode**	Enhancing cellular growth and protein expression	Require large volumes of media and cell-medium separation devices that impact negatively on cellular growth rate, protein expression and production cost.

## 4 Recombinant DNA technology applied to different host systems for VLP vaccine production

Bacterial systems (mainly *E. coli*) are used to produce approximately 28% of all VLPs (43). In these systems, viral genes are codon-optimized for bacteria and cloned into commercial plasmids with strong promoters to generate higher yields and easy initial purification (44). However, a major drawback of these systems is their inability to induce post-translational glycosylation in the eukaryotic proteins and the endotoxins created during production of recombinant proteins. Little progress has been made in introducing glycosylation into *E. coli*-based cell-free systems. Asparagine-linked glycosylation is the most general and universal PTMs that determines the folding, stability, and catalytic activity of a recombinant protein. Protein glycosylation relies on the activity of pglB oligosaccharyltransferase (Pilin glycosylation B OTase), which transfers oligosaccharides to N-linked asparagine, thereby facilitating glycosylation (45, 46). In *Campylobacter jejuni*, a single pglB gene encodes all enzymes required for the biosynthesis of N-linked glycans. The promiscuity of *C. jejuni* PglB OTase has exhibited efficiency in *E. coli* (47). Subsequently, a glycoengineered *E. coli* strain, CLM24, was developed to express both Cj PglB OTase and Cj lipid-linked oligosaccharides (48, 49). The glycosylation components of the cell-free glycoprotein synthesis (CFGpS) system do not require a purification step and thus are easy to use and cost-efficient (48). The CFGpS system is the first prokaryotic cell-free system capable of cell-free transcription, translation, and glycosylation of proteins. Recently, a glycolic platform was developed that facilitates the glycosylation of VLPs in the *E. coli* cytoplasm (50). This glycolic platform uses the asparagine glucosyltransferase of *Actinobacillus pleuropneumoniae*, which is expressed in *E. coli* and catalyzes the transfer of single beta-linked glucose onto recombinant proteins. Similarly, to overcome the drawbacks of endotoxins, several genes such as LPS that participate in the expression of endotoxins are interrupted to eliminate endotoxin contamination and produce better recombinant proteins in *E. coli*, as shown in the case of the production of a 9-valent HPV vaccine candidate in *E. coli* ER2566 strain using a chromosomal integration approach, with markedly reduced residual endotoxin levels (51).

Insect, yeast, and mammalian platforms have also been used for the production of VLPs since the 1980s (43). Recombinant insect cells are a promising alternative platform to the baculovirus–insect cell system because target proteins can be produced simply by culturing the cells (52). For example, the secretory form of influenza VLPs consisting of hemagglutinin (HA) and matrix proteins was successfully produced by recombinant insect cells (53). Manipulating genes in yeasts is comparatively straightforward, and transformed cells can easily grow to extremely high densities. Therefore, VLPs can be produced in large volumes using commercial-scale fermenters. Among the many yeast species, *Saccharomyces cerevisiae* and *Pichia pastoris* have emerged as the most promising candidates for developing VLP-based vaccines. Noticeable changes in the production of VLP-based vaccines involve recombinant strategies that induce the secretion and production of multilayered VLPs, which are typically composed of more than one type of structural protein. For example, co-expression of four structural proteins in yeast expression systems effectively produces enterovirus 71 (EV71) VLPs (54). EV71 causes hand, foot, and mouth disease (HFMD), which is associated with severe neurological complications. VLP-based vaccines have shown great potential for preventing EV71 infections. Moreover, there have been a number of attempts to produce VLPs using new expression hosts, such as *Hansenula polymorpha* (methylotrophic yeast) is particularly striking (55). *H. polymorpha* has several unique characteristics such as thermostability, avoidance of hyperglycosylation, and use of several carbon sources (56). This platform has been established for the cost-effective production of VLPs at a larger scale (40 mg/100 g) (57).

Several mammalian cells, such as Chinese hamster ovary (CHO), HEK293, amniotic fluid CAP-T, and Vero cells, are used to produce VLPs (2, 14). These cells can produce complex and accurate post-translational modifications, and therefore, are preferred for the production of complex eVLPs containing multiple structural proteins. CHO cells are not human-derived; thus, compared with other mammalian cells, they have the advantage of a lower risk of contamination by human viruses. However, low yield (0.018–10 μg/mL) (14), difficulty in scale-up, and high cost are several factors that need attention. It has been shown that the CHO‐derived viral‐like particles for hepatitis B include a combination of glycosylated and non‐glycosylated HBsAg proteins, similar to those in patients’ sera. Examples of CHO‐based vaccines are GenHevac B^®^ and Sci‐B‐Vac™. These vaccines contain the HBsAg small and middle proteins (GenHevac B) or middle and large proteins (Sci‐B‐Vac) (58, 59). Similarly, hantavirus VLPs have been designed to co-express the nucleocapsid protein and glycoproteins (Gn and Gc) in CHO cells. These VLPs produce both humoral and cell-mediated immune responses against hantavirus (60). However, CHO-derived VLPs may be contaminated with HCPs. Certain HCPs, if not removed during subsequent purification processes, have been shown to induce immunogenic responses in patients. Others can shorten the shelf life of VLP vaccines *via* various mechanisms, including polysorbate degradation. Anti-CHO antibodies have been identified in 54% of individuals with no history of exposure to protein therapeutics (61). Therefore, a reduction in HCPs to minimal levels is required. While most HCPs are removed during downstream purification steps, certain ‘difficult-to-remove’ HCPs often remain (62). Additionally, stable transfection of mammalian cells is time-consuming, transient transfection has been used to produce VLPs in several studies. For example, Wu et al. demonstrated the effective production of influenza VLPs resembling original viruses in size, structure, glycosylation, and host factor composition (63). Similarly, severe acute respiratory syndrome coronavirus-2 (SARS-CoV-2) VLPs was constructed by co-expressing four SARS-CoV-2 structural proteins in Vero E6 cells making it stable and unified, as compared to those from HEK293T cells.

## 5 Technical challenges in VLP production

### 5.1 Challenges in the transduction related parameters

Cell concentration at infection (CCI), multiplicity of infection (MOI), and time of harvest (TOH) in the baculovirus system strongly influence protein expression and, consequently, assembly of VLP. 72–120 h post-infection is considered the optimal time of harvest. Outside this period, various parameters can hinder productivity, such as cell lysis, protein degradation, and difficulties in downstream processing owing to the presence of contaminant proteins, hosts, or viral DNA. Incomplete VLP may also be released into the medium along with target mature VLP and can hinder the downstream processing of VLP (64–66). Differences in volumetric productivity, either due to low or high MOI, can be counterbalanced by modulating CCI. However, CCI is inversely correlated with productivity. Although perfusion or fed-batch techniques may overcome these limitations, they are neither practical nor economically feasible. Therefore, the development of novel techniques for process optimization is required.

### 5.2 Challenges in VLP assembly

The assembly of VLPs involves assembling single or multiple structural capsid proteins in appropriate production hosts (*in-vivo* assembly) or in cell-free conditions (*in-vitro* assembly). The nature of the stabilizing contacts can influence the VLP assembly route (67). Protein-protein interactions protect the viral genetic material from the degradation and facilitate the establishment of a relatively stable carrier/delivery system. Similarly, viral genomes coevolution with their own structural elements has resulted in interactions in which nucleic acids are the primary driving mechanism for VLP formation. For example, the formation of HCV VLP is dependent on the interaction of highly basic N-terminal 120 (68)or 124 (69) amino acids of the core protein with a 5′ untranslated region containing expected secondary structural elements. The assembly and disassembly of VLPs may also depend on the ionic or reducing conditions in which they are assembled. In more complex viruses, scaffolding proteins play an essential structural role in ensuring correct assembly of large viruses. For example, P22a is a critical scaffolding protein in the particle assembly of the herpes simplex virus (70). Assembly is not usually a single high‐order reaction, but can sometimes include a cascade of low‐order intermediate reactions. These intermediate reactions at various phases of assembly can be examined or trapped by mutagenesis of structural proteins and their expression in a suitable system. The uniformity and self-assembly of VLPs, along with their ability to withstand chemical modifications primarily on the outer surface, make them flexible and stable alternatives to nanoparticles, such as liposomes and metal assemblies (1, 8).

Self-assembly of VLPs is driven by interactions between capsid proteins and often with the viral genome. For example, approximately 25% of the HBsAg monomers in the RTS,S (Mosquirix™; the first vaccine for malaria) are genetically fused to the truncated circumsporozoite protein (CSP) and serve as protein carriers. The region of the CSP included in the RTS,S vaccine includes immunodominant B-cell epitopes (last 18 four amino acids (NANP) repeats) and three known T-cell epitopes: a highly variable CD4+ T-cell epitope before the Thrombospondin (TSP)-like domain, a highly variable CD8+ T-cell epitope within the TSP-like domain, and a conserved “universal” CD4+ T cell epitope at the C-terminus. These epitopes of Mosquirix enhanced antigen presentation to the immune system and likely facilitated strong anti-CSP antibody and T-cell responses measured in vaccinated individuals. Mosquirix has received approval from the regulatory authorities (71, 72).

To obtain superior assembly effects, cell-free or *in vitro* assembly of VLPs requires control of variables such as temperature, pH, and specific assembly solution (73). The assembly of VLPs *in vivo* necessitates a suitable thermodynamic environment for the host, which might result in the formation of malformed structures and pose technical challenges in VLP assembly and production. In this context, thermodynamic studies may be useful for identifying the most suitable environmental conditions for production during upstream processing as well as for maintaining stability throughout downstream processing (DSP) and storage. Various studies have demonstrated the effects of several physicochemical parameters (pH, ionic strength, temperature, and correct stoichiometric ratio) on the formation of VLPs (74, 75). When optimal conditions are not available, VLP destruction increases considerably. Additionally, owing to their large structure, VLPs may occasionally lose their self-assembling property or cause protein misfolding, resulting in the formation of defective VLPs (76). Time is another factor that renders antigen assembly laborious. Therefore, optimization is essential for appropriate VLP assembly and should be investigated further.

### 5.3 Challenges in upstream processing

Several process parameters, such as dissolved oxygen concentration, pH, temperature, agitation rate, inlet gas flow, and composition, influence VLP production and pose technical challenges either by hindering the cell growth or metabolic state of the cells or by interfering with the mechanisms responsible for post-translational modification. Limited or excess oxygen induces proteases that can degrade the product of interest (77). When oxygen-derived free radicals are formed owing to a high concentration of dissolved oxygen, they either induce oxidative stress in cells or cause oxidative damage to proteins (78). Temperature is another parameter that can control oxygen solubility (79).

The use of headspace aeration (bubble-free systems) or nonionic copolymers may provide a solution depending on the degree of sensitivity of the cells (77, 79–81). Cellular damage can be eliminated in a bubble-free system, but productivity decreases due to inadequate levels of dissolved oxygen. However, nonionic copolymers do not interfere with dissolved oxygen. These copolymers lower the surface tension of the culture medium and increase cell membrane rigidity, thereby inhibiting the attachment of cells to bubbles and increasing cell resistance to hydrodynamic forces.

### 5.4 Challenges in downstream processing of VLPs

The separation of a complex mixture of proteins from VLP using transduction is challenging because of their similar size and molecular weight. The same problem does not exist in the case of transfection, which thus, significantly decreases the complexity of downstream processing. Serum-free medium further improves or facilitates downstream processing because it does not contain any animal-derived supplements. Controlling various process conditions, such as optimal salt, pH, and ion concentrations, can reduce or avoid protein aggregation, which is a major challenge in downstream processing. However, aggregation during large-scale production of VLPs, owing to their larger size, leads to the loss of a considerable portion of the target protein or reduced immunogenicity (82). In a recent study, a new membrane chromatography procedure was developed to produce nano-VLPs against Ebola. This method reduces VLP size to a more manageable range, where it can not only retain its temperature stability and antigen structure, but also stimulate immunity in mice. In addition, lyophilization can be used to increase its thermostability. Lyophilization and freeze-drying are used worldwide. However, they are especially effective for tropical countries that do not have a reliable “cold-chain” of refrigeration (83). A patent was filed for the methodology against the Ebola virus in 2015 (US20170274063A1); however, in 2017, it was abandoned. Tangential flow filtration, gel permeation, ion exchange, size exclusion chromatography, and mixed-mode chromatography, including the use of improved disposable membrane technologies, can improve the productivity of purified VLP (84–86).

### 5.5 Challenges in biochemical and biophysical characterization of VLPs

The biochemical characterization of VLPs is usually performed using various techniques such as sodium dodecyl sulfate (SDS)- polyacrylamide gel electrophoresis, western blotting (WB), enzyme-linked immunoassay (ELISA), and bicinchoninic acid (BCA) protein quantification assays. Flow cytometry is now being exploited as a tool for the study and validation of VLPs (87). However, these techniques cannot differentiate between unassembled and partially or completely assembled VLPs and are time-consuming, have low sensitivity, and require high sample volumes. This leads to either the over-or underestimation of proteins, compromising downstream processing. Some of the issues mentioned above have been effectively visualized and addressed using Transmission electron microscopy (TEM). Particle size analysis using light-scattering techniques is commonly adopted to measure the hydrodynamic size and homogeneity (polydispersity) of VLPs in solution. Additionally, orthogonal methods, such as high-performance liquid chromatography (HPLC), SDS-capillary gel electrophoresis, MALDI-TOF MS, and capillary zone electrophoresis, have been developed to overcome the limitations posed by SDS-PAGE, WB, ELISA etc. Two-dimensional fluorometry is an optical fiber-based technology that can monitor several compounds, either present outside or inside cells. Therefore, *in situ* monitoring of the kinetics of VLPs is possible using this technology (88–93).

### 5.6 Challenges in critical quality attributes in VLP production

Critical Quality Attributes (CQAs), such as the biophysical, biochemical, structural, and immunochemical properties of VLPs, are critical parameters in developing vaccine candidates with good stability and delivering consistent products for the preclinical and clinical stages. In addition, these parameters also help in post-licensure life-cycle management to support market demand by upgrading the process or scale-up.

Various methods are used to characterize the physical and structural attributes of VLPs, such as transmission electron microscopy (TEM), Atomic force microscopy (AFM) for morphology, size-exclusion chromatography-high performance liquid chromatography (SEC-HPLC) for monodispersity (94), dynamic light scattering (DLS), cloud point, surface plasmon resonance (SPR) to check propensity of aggregation and circular dichroism (CD) (95) and differential scanning calorimetry (DSC) to check thermal stability (96, 97). However, it should be noted that drying of samples is required during TEM or AFM, which can introduce artifacts caused by the dehydration of VLPs. Cryo-EM can overcome the drawbacks of TEM and AFM because it preserves the VLP structure in its native form. However, cryo-EM is only useful if the VLP preparation is sufficiently homogeneous, although a pleomorphic nature can also be observed. Similarly, the AFM in the solution also reduced the drying artifacts. Another major challenge in VLP characterization is accurate measurement or quantification of VLPs to derive an accurate dose. Nanoparticle tracking analysis (NTA)/tunable resistive pulse sensing (TRPS) (98), electrospray-differential mobility analysis (ES-DMA) (99, 100) and asymmetric flow field flow fractionation with multi-angle light scattering detection (AF4-MALS) (100, 101) have been used to accurately measure the size distribution accurately. These methods are cost-efficient compared to TEM and are used to quantitatively monitor batch consistency for new vaccines.

Activity-related attributes are also important for checking the structure–function relationship in VLP-based vaccines or the integrity of key epitopes. Tools such as bilayer interferometry (BLI) and SPR are used to check epitope-specific antigenicity and epitope mapping (102). *In vitro* relative potency or mouse potency assays are used to test the release and stability of VLPs. These assays are required to define the quality and quantity of epitopes on recombinant antigens. Therefore, they play a major role in the implementation of process improvements or scale-up.

Additionally, expressing VLPs in a suitable host to provide an appropriate environment and post-purification disassembly/reassembly are essential for enhancing the stability, homogeneity, and immunogenicity of VLPs. For example, HBsAg particles contain 30% lipid by mass; therefore, a suitable host that can provide an appropriate lipid composition should be chosen. Similarly, HPV VLPs derived from *E. coli* are heterogeneous in nature because of the many ill-formed or incomplete particles. This heterogeneity can be reduced by post-purification disassembly followed by reassembly, as reported previously (97).

Demonstration and maintenance of comparability between different lots of preclinical/clinical-grade materials and between small-scale preclinical and large-scale clinical trial materials is a regulatory requirement. Process changes are implemented based on the lot-to-lot comparability exercise, an important component of chemistry, manufacturing, and control (CMC) batch analysis. Lot-to-lot comparability relies on the physical and functional properties of the vaccine, which are responsible for predicting the consistency of batches across different lots. This analysis ensures that vaccine lots are equivalent in potency, purity, and physicochemical integrity. Any process modification and formulation change between these phases must also be verified by CQA-based comparability, which is required for Good Manufacturing Practice (GMP) (103).

### 5.7 Challenges in the production of Chimeric VLPs

Chimeric VLPs are produced by assembling the structural proteins or epitopes of different viruses. For example, in the M2-HPV model, the M2e influenza epitope was inserted into the HPV 16 L1 sequence in *Nicotiana benthamiana* plants (104). L1 is the major capsid protein of HPV. The production process for chimeric VLPs depends on various factors, such as glycosylation efficiency, intermolecular chemical bonding, steric hindrance, and cell type; therefore, it is highly unpredictable. This unpredictability may lead to the formation of heterogeneous VLPs. Adding nonpeptide antigens to chimeric VLPs is another challenge because introduced antigens cannot adopt a quaternary structure and thus lead to inappropriate folding of the protein. These epitopes are not optimally exposed; therefore, the immune responses obtained against such epitopes are often limited. Thus, a VLP pseudotyping strategy or modular molecular assembly approach is a viable solution for overcoming this drawback (105, 106). In the first step of the two-step process, native VLPs and target antigens are synthesized separately. In the second step, *in vitro* assembly of the two components is performed by either covalent or noncovalent binding, which links the target antigen to the surface of the preassembled VLP. This approach significantly enhances the combination of a full-length correctly folded target protein with VLPs. Chemical cross-linking is the most commonly used method to facilitate the binding of antigens and native VLPs. Two examples of chemical crosslinking are the M2-HPV VLP conjugate vaccines of Merck & Co, Inc. and Cytos Biotechnology AG, where the M2 peptide of influenza A is conjugated to VLP (7, 107). Another example is the chemical coupling of the receptor-binding domain (RBD) of SARS-CoV-2 to cucumber mosaic virus derived virus-like particles (CuMV_TT_VLP) by a Swiss biotech company, Saiba-AG (108). These CuMV_TT_ VLPs incorporate a universal T**-**cell epitope derived from the tetanus toxin. In addition, these VLPs package bacterial RNA, which is a ligand for Toll-like receptor (TLR) 7/8 and serves as a potent adjuvant by engaging these innate receptors in specific B cells (108).

### 5.8 Challenges in maintaining stability of VLPs

VLPs can become very unstable and tend to phase separately in solution. The stability of VLPs is essential for producing viable antigens for VLP-based vaccine candidates. This is only possible after obtaining a stable formulation of VLPs. Therefore, preserving critical epitopes on VLPs, along with immunogenicity, is equally important. The introduction of nonionic surfactants into HPV VLP aqueous solutions significantly enhanced their stabilization and reduced aggregation due to heat stress and physical agitation. Generally, eVLPs are more susceptible to external environmental conditions than non-enveloped VLPs because of various factors, such as temperature changes, shear stress, fluid dynamics, agitation rate, chemical treatment, and dissolved oxygen, all of which affect the integrity and stability of VLP particles. Several modifications have been made to improve the thermostability of the eVLPs. A typical procedure involves insertion of stabilizing mutations. A study discovered (109) that inserting a stabilizing mutation in the coat proteins of poliovirus type 3 VLPs increases their stability when compared to wild-type VLPs. Because adjuvants are primarily used to enhance the stability of VLPs, proper dissolution conditions for the removal of adjuvants are also required for morphological and antigenicity analysis of VLPs. *In-vitro* relative potency (IVRP) or mouse potency under proper dissolution conditions to fully recover the antigen is used to assess the stability of vaccines over many months (110).

Another important parameter is to define the stability of VLPs in three storage conditions: real-time (long-term), accelerated, and stress (high-temperature) conditions (111). Long-term stability studies define the conditions needed for vaccine preservation and expiration date. Accelerated and stress condition tests are used to define the stability of the vaccine in the short-term deviation from preservation and extreme conditions, respectively. However, defining stability under these conditions is challenging because a conceptualized matrix is not available for VLPs owing to their non-linear nature in response to thermal and other external factors and may be very specific to the nature and composites of VLP. The stability tests include identity, appearance, volume of fill, pH, osmolarity, adjuvant content, detergent content, sterility test, endotoxin, impact on potency, and toxicity (111).

Similarly, to avoid the freezing–de-freezing cycle, critical attention should be paid to choosing appropriate and compatible cryoprotectants to preserve the native structure of VLPs.

## 6 Recent advances to enhance productivity and immunogenicity of VLP expression systems

### 6.1 Productivity

Several purification steps, such as ion exchange, size-exclusion chromatography, and ultracentrifugation, are used to obtain purified VLPs. Furthermore, HCP are removed by polishing. This step is followed by sterile filtration and formulation to obtain a safe and effective vaccine. At the industrial scale, size-exclusion chromatographic purification or sucrose gradient ultracentrifugation steps are used, which increase production costs. Many scientists are working on replacing these steps with more robust purification steps. For example, a robust purification protocol obtained approximately 90% pure foot and mouth disease virus (FMDV) VLPs. This protocol does not include non-preferred industrial size-exclusion chromatographic purification or sucrose gradient ultracentrifugation steps (32). This approach could be used for large-scale industrial production of *E. coli*-based VLPs against FMDV infections of different serotypes, making this approach cost-effective. Carvalho et al. showed that the use of sulfated cellulose membrane adsorbers (SCMA) enhances the scalability of vaccines compared to conventional ion exchanger membrane adsorbers by reducing the number of purification steps. This study suggested that SCMA could qualify as a generic platform for purifying VLP-based influenza vaccines (112). However, if the same techniques can be used to produce several different VLPs, it has not been tested and needs further investigation.

Similarly, a chromatographic procedure was used to purify HIV-1 gag VLPs using a mammalian platform, in which the clarified and filtered cell culture supernatant was directly processed on an anion-exchange monolith. Gag, or group-specific antigen, is the major structural protein of HIV-1 and all other retroviruses, and comprises approximately 50% of the mass of a viral particle. This method showed a 220-fold improvement in productivity compared to density gradient centrifugation (35). Plants such as tobacco and related *Nicotiana* species are commonly used to generate plant-made pharmaceutical proteins (PMPs). However, these plants have high levels of phenolics and toxic alkaloids; therefore, the downstream processing of target PMPs is hindered by technical and regulatory difficulties. Hence, lettuce-based production systems are used instead of tobacco-based platforms because of their low levels of secondary metabolites (113). More complex VLPs have production challenges owing to the incorporation of envelope proteins or their stable nature. Therefore, new technologies must be developed. For example, in the case of enveloped HIV VLP, crossflow filtration across a hollow-fiber cartridge membrane under low-stress conditions concentrates the desired material with 95% recovery.

### 6.2 Immunogenicity

VLPs have intrinsic immunostimulatory properties because of their ability to activate antigen-presenting cells directly and therefore have great potential for use as antigenic platforms (114). This immunogenicity can be further improved and oriented by incorporating non-coding RNAs (ncRNAs) that act as TLR7/8 ligands. For example, the incorporation of ncRNA into murine leukemia virus (MLV)-derived VLPs provides further TLR-mediated activation and Th1-type CD4+ and CD8+ T-cell response orientation (115). TLRs are expressed by innate immune cells, predominantly antigen-presenting cells (APCs), and recognize various pathogen components. Upon binding to PAMPs, TLR signaling activates APCs, which leads to the production of cytokines, increased co-stimulatory molecule expression, and an enhanced capacity to present and cross-present antigens on MHC class I molecules (116).

The expression of antigens either in their core or on the surface of VLPs significantly improves their immunogenicity, favoring the induction of both B and T-cell-mediated immune responses. Compared to non-enveloped VLPs, eVLPs have the added advantage of presenting the surface proteins of enveloped viruses in their native form. These features can enhance both the humoral and cell-mediated immune responses. For example, the gag protein of Moloney MLV in HEK-293 cells was cleaved to generate a group of proteins that function to create the structured inner core of a VLP. These include matrix, capsid, and nucleocapsid proteins. The capsid protein is the main structural element of a mature virus particle, forming the core-shell around the nucleocapsid-RNA complex, whereas the matrix protein remains linked to the viral lipid bilayer. The commercial name of this vaccine is VBI-1501, which showed high immunogenicity in animals and was used in a phase I clinical trial. Another eVLP vaccine candidate, VBI-1901, is undergoing phase II testing for therapeutic immunization against glioblastoma. Similarly, two other vaccines, VBI-2902a and VBI-2905a, are eVLP-based COVID-19 vaccines for Wuhan and B.1.351 (Beta) variants of SARS-CoV-2, respectively. These vaccines contain a stabilized form of the spike protein of SARS-CoV-2 inserted into the eVLP lipid bilayer (117, 118).

The immunogenicity of VLPs can be increased by engineering VLPs, allowing for a more versatile display of antigens (119). To maximize versatility in displaying foreign antigens of varied compositions, sizes, and structures, the recombinant fusion approach is adopted. In this approach, foreign antigens are inserted into sites inside the viral structural protein to display them on the surface of the resulting VLP’s. However, because peptide insertions into viral capsid proteins frequently result in protein-folding failures, peptide insertions frequently impact the ability of recombinant proteins to assemble into VLPs. To avoid these failures, preformed VLPs might be employed as scaffolding. Foreign antigens are attached to the VLP scaffold using various conjugation techniques (120–123). Similarly, the magnitude and longevity of immune responses of VLPs can also be enhanced by modifying VLPs in such a way that their interactions with specific receptors on or within immune cells (124), interactions with molecules that regulate trafficking (125), and compatibility with adjuvants can be increased. Many VLPs have molecular and structural properties that can spontaneously stimulate the immune system without adjuvants. Nevertheless, the use of adjuvants in VLPs vaccine formulations may enhance immunogenicity and stimulate a specific immune response.

**Table 4** summarizes the most recent methods for increasing VLP productivity/immunogenicity.

**Table 4:** Strategies to enhance productivity/immunogenicity of VLP.

Platform	Disease	Strategy	Reference
Mammalian	Dengue	Epitope tagging and one step affinity chromatography	(126)
Plant	Zika Virus	VLP displaying the envelop III (EIII) domain of ZIKV on the Hepatitis B core antigen; easy production and purification	(127)
Mammalian	HIV	Chimeric C1q (first subcomponent of the C1 complex involved in the classical pathway of complement activation and ligand of CD91)/CD40L/HIV VLP to bind, cross the epithelial layer, access and activate the sub-mucosal layer DC, and ultimately induce enhanced mucosal and systemic immune responses.	(128)
*E. coli*	COVID-19	Cucumber mosaic virus derived-receptor binding motif (CuMV_TT_‐RBM) VLPs incorporate a universal T‐cell epitope derived from tetanus toxin (TT). The newly developed platform enhances the interaction between Th cells and B cells and is expected to improve responses in elderly individuals who are often less reactive to vaccines.	(41)
Baculovirus	Rabbit hemorrhagic disease virus (RHDV)	VLPs displaying two model B-cell epitopes at different surface-exposed insertion sites	(129)
*E. coli*	Hepatitis B	99% DNA impurities were removed using POROS 50 HQ chromatography (Applied Biosystems). POROS HQ is based on a quaternized polyethyleneimine functional group yielding a high capacity. In Perfusion Chromatography technology both enzymes and affinity ligands are immobilized on POROS media. In this technology, rapid on-column protein digestions with rapid on-column immunoassays and chromatographic separations are combined, which helps in creating new assays with unlimited potential.	(130)
Mammalian	COVID-19	Inclusion of c-GMP in VLP, which enhance immunogenicity	(131)
*E. coli*	Foot and Mouth Disease	Four Simple Biomimetic Mineralization Methods to Improve the Thermostability and Immunogenicity of Virus like Particles	(132)
*E. coli*	Japanese encephalitis	B and T cells epitopes on VLP	(133)
Pichia Pastoris	Hepatitis B	Replacement of alcohol oxidase 1 (AOX1) structural gene with a construction composed of the AOX1 promoter–HBsAg gene expression cassette	(134)
Pichia Pastoris	hand, foot and mouth disease	Coxsackievirus A6 (CA6) VLPs were produced in *Pichia pastoris* yeast transformed with a vector encoding both P1 and 3CD proteins of CA6. CA6-VLPs consisted of VP0, VP1, and VP3 capsid subunit proteins, which are generated by the cleavage of P1 by 3CD proteins	(135)
S. cerevisiae (D-crypt™)	COVID-19	Co-expression of spike, membrane, and envelop proteins	(136)
E. coli	Influenza	Packaged RNA	(137)

## 7 VLP-based vaccines in clinical and preclinical stages

Several VLP-based vaccine candidates tested in clinical and preclinical trials are summarized in **Tables 5A**, **B**, respectively. Several VLP-based vaccines have been developed against SARS-CoV-2, five of which are currently undergoing clinical trials. The main antigenic component of these vaccines is the spike protein, specifically the RBD, which is involved in viral entry and antibody neutralization. By displaying multiple full-length spike proteins or only RBDs on the VLPs surfaces, especially on eVLPs, these vaccines have shown high immunogenicity in clinical trials, generating neutralizing antibodies and inducing cell-mediated responses. However, introducing additional structural proteins as antigens in eVLP-based vaccines may lead to more complex and native-like structures and generate broad-spectrum immunity against various SARS-CoV-2 variants (3, 146).

**Table 5A:** VLPs based vaccine candidates in clinical trials.

Platform	Disease/Vaccine name	Manufacturer	Commercially available	Clinical Trial Status
Bacteria	Malaria (MalariVax, ICC-1132	New York University School of Medicine (Apovia)	No	Phase I (138) (NCT00587249
	Allergic rhinitis and asthma (CYT003-QβG10a) (139)	Cytos Biotechnology	No	Phase II (NCT00924105
	HPV infection (HPV6/11) (43)	Beijing Wantai Biological Pharmacy Enterprise Co., Ltd (Beijing, China)	No	Phase II (NCT02710851
	Influenza (ACAM-FLU-Aa)	Sanofi Pasteur (140)	N	Phase I (NCT00819013)
	Cervical cancer (HPV 16/18 vaccine)	Xiamen Innovax (141)	No	Phase III (NCT05045755
	HPV infection and cervical cancer (HPV 16/18 vaccine)	NCI (142)	No	Phase IV (NCT05237947)
Yeast	DTaP, Hepatitis B, Poliovirus, and Haemophilus b combination vaccine is an active immunizing agent used to prevent infections caused by diphtheria, tetanus (lockjaw), pertussis (whooping cough), hepatitis B virus, poliovirus, and Haemophilus influenza type b (Hib) bacteria (Hib-DTP-Hep B vaccine) (143)	P.T. Bio Farma	No	Phase III (NCT04071379)
	COVID-19 (PRAK-03202) (136)	Premas Biotech Private Limited, India	N	Phase I
	Oral COVID-19 (Oravax)	Oramed	N	Phase I (Imagine Oral Vaccines - Oravax Medical (ora-vax.com))
Insect	H1N1 2009 pandemic influenza	Novavax (Phase II) (144)	NA	Phase II (NCT01072799)
	Erythema infectiosum, transient aplastic crisis (Parvovirus B19 vaccine)	Meridian Life Science, Inc (Phase I) (145)	N	Phase I (NCT00379938-Terminated)
	COVID-19 (SARS-CoV-2 VLP vaccine)	Radboud University (146)	No	Phase I (NCT04839146)
	COVID-19 (SARS-CoV-2 VLP vaccine)	The Scientific and Technological Research Council of Turkey (146)	No	Phase II (NCT04962893)
	Encephalitis (Venezuelan Equine Encephalitis Monovalent Virus-Like Particle Vaccine) (VEEV)	SRI International	No	Phase I (NCT03776994)
Mammalian cells	Hepatitis B/HIV (GenHevac B^®^)	ANRS, Emerging Infectious Disease (147)	NA	Phase III (NCT0067083), Phase III (NCT00480792)
	Malaria, (RTS, S/AS01 Mosquirix™)	METU and Bilkent University, Nobel Pharmaceuticals	No	Phase I/II (NCT01883609) Phase III (NCT03143218)
	Chikungunya (PXVX0317)	Emergent BioSolutions (148)	No	Phase III (NCT05072080) Phase I
	COVID-19 (VBI-2902a)	VBI Vaccine (149)	No	(NCT04773665)
Plant cells	n-COVID-19 (CoVLP+AS03 vaccine or Coronavirus-like particles (CoVLP) +Adjuvant System 03)	Medicago lnc (150).	NA	Phase III (NCT05040789)
	Influenza/quadrivalent, recombinant, virus-like particle influenza vaccine (QVLP)	Medicago lnc (151)	NA	Phase I (NCT01302990), Phase II (NCT02768805) (152), Phase III (NCT03321968)

**Table 5B:** VLP based vaccines at the preclinical phase.

Platform	Production Yield (14)	Virus/Disease	Adjuvant	Animal	Dosage	Humoral immune response	Cell mediated Immune response	Company /Organization
**Yeast**	0.75 to 700 μg of protein per ml of culture	Dengue virus/Dengue	Alhydrogel	Mice, Rhesus macaques	20 µg	High	NA	ICGEB-Sun Pharma, India (153, 154)
		Nervous necrosis virus/viral nervous necrosis in marine fish	Mineral Oil	Larger sea bass	80 µg	High	High	Technical University of Denmark (155)
		Japanese encephalitis virus/epidemic encephalitis	Non-adjuvanted	Pigs	2.5, 5, 10, and 20 μg	High	High	Shihezi University (156)
		Norovirus/ Gastroenteritis	Alhydrogel	Mice	3 μg	High	NA	Anhui Zhifei Longcom Biopharmaceutical Co., Ltd (157)
**Insect(sf9) (** 158)**/Mammalian** (159, 160)		HCV/Hepatitis	Vary as per platform	Mice, Rabbit and Chimpanzee	Vary as per platform	Insect: Denatured VLP: Low Live VLP: High Mammalian: High	Low High High	Indian Institute of Science, Bangalore NIDDK, USA
**Mammalian cells (** 161)	0.018 and 10 μg/ml	Chikungunya virus/Chikungunya	dsRNA Adjuvant	Mice	10^8^ IUs	High	NA	Vaxart, Inc, CA USA (161)
		Powassan virus/encephalitis, meningitis, and meningoencephalitis	Squalene-based oil-in-water nano-emulsion (AddaVax)	Mouse	1, 5, or 10 µg	High	Th1 mediated response	American Type Culture Collection (162)
		HIV/Acquired immune deficiency syndrome (AIDS)	Adjuplex adjuvant	Rabbit	250 and 500 µg	High	High	National Microbiology Centre, Madrid, Spain (163)
		HCV/Hepatitis	AddaVax adjuvant	Tricolor guinea pigs	10 and 40 µg	High	Not evaluated	Burnet Institute, Melbourne, Australia (164)
		Dengue Virus/Dengue	Titer Max Gold	Mice	10 µg	High	Not evaluated	Hamamatsu University School of Medicine, Hamamatsu (126)
**Bacteria (** 165)	0.75 to 700 μg of protein per ml of culture	Zika virus/Zika		Mice		High	NA	University of Oxford, Oxford OX1 2JD, UK
		Porcine reproductive and respiratory syndrome virus (PRRSV)/Porcine reproductive and respiratory syndrome	ISA201 adjuvant (SEPPIC, Paris, France)	Pigs	100μg	Low	NA	Jingzhou Changxin Biotechnology Co., Ltd (166).
		**Japanese Encephalitis Virus/** **Encephalitis**	Complete Freund’s adjuvant	Guinea Pigs and Mice	50 µg	High	NA	Chinese Academy of Agricultural Sciences, Shanghai (133)
		Porcine pestivirus/ Congenital tremor (CT) type A-II	Freund’s complete adjuvant	Mice	Single dose	High	NA	South China Agricultural University, Guangzhou (167)
		*Chlamydia trachomatis*/ Infection	Non-adjuvanted	Mice	5 µg	High	NA	Mount Holyoke College, 50 College St., South Hadley, MA (168)
		Dengue Virus/Dengue	Non-adjuvanted	Mice	5 µg	High	NA	University of New Mexico Health Sciences (169)
**Insect(sf9) (** 170)	0.2 and 18 μg/ml	Coxsackievirus/Hand, foot and mouth disease (HFMD)	Freund’s adjuvant	Mice	10 μg	High (elevated)	NA	Guilin Medical University (171)
		HEV/Hepatitis-E	Not-used	Pigs	100 μg and 200 μg	High	NA	Konkuk University (172)
		Avian influenza virus/ influenza	ISA VG70 oil adjuvant	Chickens	512 HAU (hemagglutination units)	High	Th1 type	Komipharm Institute (173)
		Plasmodium berghei/Malaria	Non-adjuvanted	Mice	150 µg	High memory B cell response	High	Kyung Hee University (174)
		H1N1 seasons influenza virus/Influenza	Non-adjuvanted	Mice	10 µ	High memory B cell response	High	Kyung Hee University School of Medicine (175)
		H5N1/H7N9 Avian Influenza Virus/Influenza	Montanide ISA 71R VG	Chickens	7.5 µg	High	NA	Yangzhou University, Yangzhou, China (176)
**Plant**	4 to 2380 pg/mg of leaf	Norovirus/ Gastroenteritis	Non-adjuvanted	Rabbit	200 μg, 300 μg, and 400 μg	High Th2 bias response	Low	Denka Company (177)
		West Nile virus (WNV)/ Encephalitis	Montanide Gel adjuvant	Mice	5 µg	High	NA	University of Cape Town, Cape Town (178)

## 8 Conclusion

Currently, only a few VLP-based vaccines are commercially available against various pathogens such as zika virus, HCV, HBV, and HPV due to various hurdles in their development processes. Therefore, these hurdles must be addressed to enhance commercialization. Researchers are trying to refine VLP-based vaccines at the formulation level by selecting appropriate adjuvants and stabilizers/protectants, administration routes, and delivery vehicles, such as liposomes, poly(lactide–co-glycolide) microparticles, or alginates to reduce the number of vaccine shots and dosage. Concomitantly, methods that can enhance the shelf life of vaccines and avoid cold chains are under investigation.

From an economic point of view, Gardasil is the most successful VLP-based vaccine, yielding over $48.7 billion in 2021. Gardasil has been replaced by Gardasil9 that is an adjuvanted non-infectious recombinant 9-valent vaccine. Gardasil9 has increased concentrations of L1 VLPs for HPV 16 and 18 in order to induce antibody responses in comparison to Gardasil (179). L1, is a ~55 kDa protein with the ability to spontaneously self-assemble into VLPs. In contrast, in other VLP based vaccines generated in the last three years, such as Cervarix for HPV and Engerix-B, Recombivax HB, and Hepavax-Gene for HBV, the revenue has not been reported. Cervarix contains purified proteins from two types of human papillomaviruses (types 16 and 18) (180). It is made using monophosporyl lipid A (MPL), a purified lipid (fat-like substance) extracted from bacteria, which enhances the immune response of the vaccine. VLPs and MPL are then fixed onto an aluminum compound to stimulate a better immune response. MPL is a heterogeneous mixture of congeneric lipid A with 4′-phophoryl groups and varying numbers of acyl chains (181). MPL adjuvant in the form of AS04 mimics a Toll-like receptor 4 agonist providing direct stimulation of antigen presenting cells, pronounced cellular and humoral immune responses, and long lasting antibody responses (179).The Hepavax-Gene (thiomersal free) and Engerix B are non-infectious recombinant hepatitis B vaccines that contain highly purified HBsAg. Engerix-B and Recombivax vaccines differ only in the concentration of HBsAg and the nature of the aluminum adjuvant. Engerix-B uses aluminum hydroxide as an adjuvant and trace amounts of thimerosal from the manufacturing process (182). On the other hand, Recombivax contains aluminum hydrophosphate sulfate. Currently, both academic and industry partners collaborate to produce cheaper vaccines that are affordable to low- and middle-income countries (LMIC). However, based on past events (e.g., HBV vaccination), this appears to not have occurred before. For example, life-saving vaccines such as the HPV VLPs Gardasil and Cervarix were first introduced at relatively high prices in industrialized countries and private sectors in developing countries. Therefore, reaching LMICs with these vaccines took longer than expected. The financial viability of biopharmaceutical companies is the main hurdle in this process and needs to be addressed. Commercial names of various licensed VLP vaccines are given in **Table 6**.

**Table 6:** Licensed VLP based vaccines.

Platform	Disease/Commercial name	Manufacturer	Commercially available	Clinical Trial Status
Bacteria	Hepatitis E (Hecolin1) (183)	Xiamen Innovax Biotech Co. Ltd., China-Licensed (184)	License in China	NCT02189603
Yeast	Hepatitis B (Engerix-B^®^) (185)	GlaxoSmith Kline (185)	Yes	NCT02901951, NCT01847430, NCT00984139
	Hepatitis B (Enivac HB) (186)	Panacea Biotec Ltd.	Yes	NA
	Hepatitis B (Gene Vac-B^®^) (187)	Serum Institute of India Ltd.	Yes	Phase I/II (NCT05326152)
	Hepatitis B (Recombivax HB^®^) (188)	Merck and Co., Inc.	Yes	Early phase I (NCT04162223), Phase III (NCT00414050), Phase II (NCT00107042)
	Hepatitis B (Revac-B+™) (189)	Bharat Biotech International Ltd.	Yes	NA
	Hepatitis B (ShanvacTM-B) (190)	Shantha Biotechnics Ltd.	Yes	Information not available
	Hepatitis B (Euvax B) (191)	LG Life Sciences	Yes	NCT01468051, Phase III, (NCT02697474)
	Hepatitis B (Heberbiovac HB) (192)	CIGB – Heber Biotec	Yes	NA (RPCEC00000109) https://rpcec.sld.cu/en/trials/RPCEC00000109-En
	Hepatitis B (Hepavax-Gene^®^) (193)	Crucell	Yes	Phase III (NCT01349283), Phase IV (NCT02713620), Phase IV (NCT02713620)
	HPV cancer/Gardasil^®^	Merck & Co (194)	Yes	Phase III (NCT03546842)
	Malaria (RTS,S/AS01 Mosquirix™) (72)	GlaxoSmithKline (GSK) and the Walter Reed Army Institute of Research (WRAIR)	Yes	Phase III (72) (NCT03143218)
Insect	HPV cancer/Cervarix^®^	GlaxoSmithKline (180)	Yes	Phase IV (NCT00534638)
	Human Norwalk Virus illness	LigoCyte Pharmaceuticals Inc	Yes	Phase I (195) (NCT00806962, NCT01435811)
Mammalian cells	Hepatitis B (Sci-B-Vac™) (59)	METU and Bilkent University, Nobel Pharmaceuticals	Yes	Phase III (NCT04531098, NCT04209400), Phase IV (NCT04179786)

## Author contributions

PK, NA, KA, and RG were responsible for the conceptualization of the manuscript. SR, AJ, RR and RG: Contributed to latest literature review and manuscript drafting. All authors contributed to the article and approved the submitted version.

## Glossary

**Table:**

VLP	Virus-like particles
eVLP	Enveloped virus-like particles
PAMP	Pathogen associated molecular patterns
DC	Dendritic cells
	
HBcAg	hepatitis B core antigen
HBsAg	hepatitis B surface antigen
HBV	hepatitis B virus
HCV	hepatitis C virus
PTMs	Post-translational modifications
HPV	Human Papilloma Virus
HIV	Human Immunodeficiency Virus
HEV	Hepatitis E virus
RVFH	Rift Valley fever virus
CFGpS	Cell-free glycoprotein synthesis system
Cj	*Campylobacter jejuni;* OTase, Oligosaccharyltransferase
EV71	Enterovirus 71
CHO	Chinese hamster ovary
CAP-T	CEVEC’s Amniocyte Production-T Antigen
CCI	Cell concentration at infection
MOI	Multiplicity of infection
TOH	Time of harvest
TSP-like domain	Thrombospondin-like domain
CSP	Circumsporozoite protein
DSP	Downstream processing
TEM	Transmission electron microscopy
EM	Electron microscopy
WB	Western blot
ELISA	Enzyme-linked immunoassay
SDS	Sodium Dodecyl Sulphate
MALDI-TOF MS	Matrix-assisted laser desorption/ionization-time of flight mass spectrometry
CQAs	Critical Quality Attributes
AFM	Atomic force microscopy
SEC-HPLC	Size-exclusion chromatography-high performance liquid chromatography
DLS	dynamic light scattering
SPR	Surface plasmon resonance
CD	Circular dichroism
DSC	Differential scanning calorimetry
NTA	Nanoparticle tracking analysis
TRPS	Tunable resistive pulse sensing
ES-DMA	Electrospray-differential mobility analysis
AF4-MALS	Asymmetric flow field flow fractionation with multi-angle light scattering detection
CMC	Chemistry, manufacturing, and Control
GMP	Good Manufacturing Practices
BLI	Bilayer interferometry
FMDV	Foot and mouth disease virus
SCMA	Sulfated cellulose membrane adsorbers
PMPs	Plant-made pharmaceutical proteins
CuMV_TT_VLP	*Cucumber mosaic virus* derived virus-like particles
APC	Antigen presenting cells
MHC	Major Histocompatibility complex
TLR	Toll-like receptor
RBD	Receptor binding domain
LMIC	Low- and middle-income countries
RBM	Receptor binding motif
RHDV	Rabbit haemorrhagic disease virus
TT	Tetanus toxin
CA6	Coxsackievirus A6
GMP	Guanosine mono phosphate
DSV4	Dengue Subunit Vaccine Tetravalent
PRRSV	Porcine reproductive and respiratory syndrome virus
WNV	West Nile virus
AddaVax	Squalene-based oil-in-water nano-emulsion
PRRs	Pathogen recognition receptors
IVRP	*In-vitro* relative potency
HPV L1-protein	L1 is a major capsid protein of type human papilloma virus
HA	Hemagglutinin
M2-HPV model	The M2e influenza epitope was inserted into the HPV 16 L1 sequence in *Nicotiana benthamiana plants;* SARS-CoV-2, Severe acute respiratory syndrome coronavirus 2
CHOEBNALT85 cell line	QMCF/CHO based stable cell line. QMCF technology utilizes genetic modifications which increase the expression vector copy-number while ensuring their even distribution during cell division, guaranteeing near-stable expression levels
HAU	hemagglutinating unit, defined as the amount of virus needed to agglutinate an equal volume of a standardized RBC suspension
EIII domain of ZIKV	Envelope protein domain of Zika Virus
C1q	C1q is the first subcomponent of the C1 complex involved in the classical pathway of complement activation and was reported to be the ligand of CD91, a multifunctional scavenger and signaling receptor
P1	Precursor protein 1
P1 and 3CD proteins of CA6	CA6-VLPs consisted of VP0, VP1, and VP3 capsid subunit proteins which are generated by the cleavage of P1 by 3CD proteins
AOX1	Alcohol oxidase I
POROS 50 HQ chromatography	It is based on a quaternized polyethyleneimine functional group yielding a high capacity, High Performance Chromatography&trade
	
	
ISA 71 R VG	A water-in-mineral-oil adjuvant based on a specific mannide oleate based surfactant system that has been designed to increase vaccines stability
Montanide ISA 70	A adjuvant composed of a natural metabolizable oil and a very refined emulsifier from the mannide monooleate family
